# Notes of a Visit to the Public Lunatic Asylums of Scotland

**Published:** 1856-01-01

**Authors:** John Webster

**Affiliations:** Physician to the Scottish Hospital, &c.


### Art. IV.—NOTES OF A VISIT TO THE PUBLIC LUNATIC ASYLUMS OF SCOTLAND.

BY JOHN WEBSTER, M.D., F.R.S., AND F.R.C.P.,

*Physician to the Scottish Hospital, &c.*

In former numbers of the "Psychological Journal," I communicated an account of various visits made to public asylums for the insane in France, which were repeated during subsequent years, although not published, from entertaining the opinion that, any additional data then collected would have too much resembled previous statements, to make them sufficiently interesting. Last autumn, my sphere of observation was changed. Then I visited Scotland, in order to inspect different institutions for lunatics in that part of the empire: as well to obtain correct information respecting their organization and management, as also to compare these national establishments with asylums of other countries. Believing an outline of my proceedings may prove acceptable, I would therefore remark that six public institutions were examined,—namely, Edinburgh, Glasgow, Perth, Dundee, Montrose, and Aberdeen: upon each of which I propose giving a brief notice, wherein it will be my object to detail facts, rather than to enunciate opinions, so that readers may thereby be enabled to form their own conclusions, regarding the different establishments thus brought under review.

However, before adverting to any of the institutions abovementioned, some notice of the laws applicable to lunatics in North Britain will not seem inappropriate, especially to members of the medical profession resident in England: many of whom, perhaps, may not be fully cognizant of the legal enactments respecting insane persons, and the administration of asylums throughout Scotland. With the view of enabling psychological jurists to study this important subject more minutely than could be given by any cursory statement on the present occasion, I would observe that the following Acts of Parliament, recently passed in reference to the insane, may be consulted advantageously; since, by these statutes Scottish lunatic establishments, or *madhouses*, according to parliamentary phraseology—but which improper designation ought to be revised—are now regulated: whilst " fatuous or furious persons, or lunatics," are taken care of and treated throughout the country. The Acts now referred to are,—1st, the 55th of George III., cap. 69; 2nd, the 9th of George IV., cap. 34; and 3rd, the 4th and 5th of Victoria, cap. 60, each of which will repay perusal.

Considering it unnecessary to discuss at any length the various clauses of the above enactments, I would for the present observe that, no person can be received into any public hospital or asylum for the insane in Scotland, without a warrant from the sheriff of the county, or his substitute: upon the petition of some relative or friend of the lunatic, which specifically states the party named therein " is in such a state of mental derangement as to require treatment in a lunatic asylum." This document must be accompanied by the certificate of some legally-qualified medical practitioner, who declares, *on soul and conscience*, that, to the best of his belief and knowledge, the patient designated is insane, and a proper person for admission. Where no legally-qualified medical practitioner can be procured to put his name to the required certificate, it will then be sufficient if signed by any medical man of character whom the sheriff may think proper to employ: not being the medical officer of the asylum to which it is proposed to send the lunatic.

Such are the chief formalities, requisite prior to the reception of a lunatic patient into any licensed house; but in reference to private individuals who take charge of single maniacs, it is enacted by the 9th of George IV., that no person shall receive into his exclusive care and maintenance, except a relative, any one insane patient, without first having an order, and certificate signed by *two* medical practitioners: a copy of which the party receiving such single lunatic must transmit to the sheriff of the county in which he resides, within five days after the party's reception, accompanied by a statement correctly designating the parish wherein the house is situated, and also the name of its occupier. Afterwards, annually, on or within seven days of the 1st of January, a certificate must be transmitted to the sheriff, signed by two physicians or surgeons, describing the then state of such insane person; and lastly, should the party die, or be removed elsewhere, these events must be forthwith notified to the sheriff. Besides the above essential formalities, any individual incurs a penalty of 50*l.* who receives into his house an insane person contrary to this enactment.

Although the clauses now specified embody the principal regulations respecting lunatic asylums, nevertheless, believing some readers of this paper might desire to possess more precise knowledge respecting the law generally, in Scotland, with reference to lunatics, particularly those who have not the Acts above quoted, or any legal publication upon the subject in their library, even at the risk of being reckoned tedious, I would subjoin the following additional remarks upon the subject: only premising, if more agreeable, the next nine paragraphs may be passed over as supererogation.

According to the several statutes previously mentioned, no one can, under a penalty of 200*l.* and expenses, keep an asylum for lunatics, without a licence from the sheriff of the county or his substitute, which must be renewed yearly: a certain sum being charged upon every mad person therein specified, both for the first granting, and each annual subsequent renewal of the licence. Further, all sums so received form part of the *rogue money* of the county, as ordered by fiat of Parliament. The last regulation seems, to say the least, a most extraordinary application of monies thus obtained. It looks as if classing the insane actually with rogues: or, like ancient proceedings, when lunatics were consigned to prisons, and there treated the same as vagabonds or criminals. Of this kind, examples were formerly too frequent: although, thanks to the present enlightened views entertained by all classes, such harsh treatment is now repudiated, and a more humane mode is happily pursued, almost universally. This reality having been acquired, even the semblance of connecting asylums with law-breakers, in any way, should be avoided; and therefore, making the proceeds obtained from granting licences to institutions for the insane form part of such a fund as county rogue money, is most objectionable, and ought to be altered f[illegible]with.

Besides being invested with other official functions, as already stated, sheriffs are directed to use all means for ascertaining whether persons placed in asylums ought to be detained therein, and to make such order for the lunatic's care, or confinement, or liberation, as circumstances require. They may also commit vagrant maniacs. Again, no person can be admitted into any institution for the insane, without a sheriff's order; whilst every individual receiving a lunatic into his house, to be treated or confined, without such authority or licence, forfeits 200*l.* and expenses, *toties quoties.* Further, every medical practitioner giving a certificate of lunacy, without having taken proper means for ascertaining the fact, forfeits 50*l.* and expenses. Lastly, asylums are inspected twice a year; once by the sheriff or his substitute, and once by the sheriff in person, with such medical man, or others, as he thinks should accompany him on that occasion.

Various books or registers ought to be kept in every licensed house, which must be produced to the inspectors, who insert the date of their inspection, together with any observations they deem expedient. The sheriff may recal any licence upon a report of two authorized inspectors. He can likewise make fitting rules for the management of asylums, and enforce the same with penalties, not exceeding 10*l.* for each offence. Houses of reception having 100 patients must have a resident physician or surgeon, or if the institution contains fewer inmates, unless kept by a physician or surgeon, it must be visited twice weekly by a qualified medical practitioner. Such visiting or resident physicians or surgeons being directed to make reports to the keeper, as he is termed at present, and once weekly to enter their remarks, signed, in a register, according to forms given in the schedule, which must be afterwards exhibited to the inspectors. An account of all monies received from asylums, and expenses incurred, is transmitted by the sheriff to the County Commissioners of Supply; besides which, he must also send a statement of the number of asylums in his county, and of the names, amount, and description of persons therein confined, to the Clerk of the High Court of Justiciary, as also to the College of Physicians at Edinburgh.

Inspectors of asylums are elected by the College of Physicians of Edinburgh, and the Faculty of Physicians and Surgeons in Glasgow: each of these bodies appointing, annually, four of their ordinary resident members to that office. From amongst these, the sheriffs of Mid-Lothian and Lanark may employ any of the so elected gentlemen, within their respective jurisdictions. In other counties, sheriffs can choose for inspectors any physician qualified to make such inspection, unless local or other circumstances render that proceeding inexpedient. Justices of the peace may also appoint, at the Michaelmas quarter sessions, three of their number to visit and inspect any private or public asylum within their own county, and to report annually "thereanent." Ministers are also empowered, with written consent of the sheriff, to visit madhouses within their parishes: although the keeper may refuse them admission, if he thinks such visit would be prejudicial to the patients; but he must always enter this refusal, and its cause, in the register. In addition to the above regulations, provision is likewise made for the procurator-fiscal of the county to enforce the acts now existing, and to recover all penalties incurred by parties contravening the law: which sums, like those received for licences, form part of the *Rogue money!*

The above constitute the principal laws in reference to institutions for the insane, and the admission or confinement of lunatics therein. Other minor regulations might be mentioned, but it is considered unnecessary. Nevertheless, before proceeding with my Notes respecting the present condition of public asylums in Scotland, I must beg permission to add to previous legal observations, that there are various degrees of aberration of intellect recognised by lawyers in North Britain. These may be divided into two classes. The first comprehends every person who is, in judicial language, *fatuous*, and naturally an idiot, or furious mad, and a lunatic; or whose external senses are so imperfectly organized, as to render the party implicated totally unfit to undertake and superintend the independent management of him or herself, or affairs. The other division includes those persons who, although not so devoid of reason as to be absolutely incapable of acting for themselves in the minor affairs of life, are yet, from imbecility or weakness of judgment, considered by the law fit subjects for a limited degree of restraint in matters of importance. The remedy in the former case, is to place the fatuous or furious person under permanent and unlimited "curatory." The proceeding in the latter example being "interdiction," as it is denominated; by which lavish and facile individuals are disabled from signing any deed to their prejudice, without the previous consent of their appointed interdictors.

To enter into any lengthened discussion respecting the above forensic questions, would be rather incompatible with the chief purpose aimed at in the present communication, therefore I will only now briefly observe, when thus bringing several important features characterizing the lunacy laws of Scotland, before readers of the "Psychological Journal," that one of the objects proposed, among others, was to notice briefly a procedure in that portion of Great Britain, which has many recommendatory reasons for its adoption, from being applicable to individuals not certainly altogether sane, but yet quite incompetent to manage their own affairs without some control. Interdiction here adverted to becomes truly a kind of *mézzo termine*, as it might almost be called; or, something like that peculiar issue designated in the criminal law of Scotland, a "non proven verdict." Upon the above legal proceeding, prevalent North of the Tweed, in reference to imbecile persons, one or two observations seem advisable, with a view to induce subsequent discussion by legists and psychological physicians.

This system of interdiction, according to Scottish legal authorities, constitutes a species of restraint provided for those who, from weakness, facility, or profusion, are liable to imposition. It is directed at the option of the judge or Lord Ordinary at Edinburgh, on proper evidence proving the facility of the person arraigned: or is voluntarily imposed by the party applying for such protection. Hence the distinction into voluntary, and judicial interdiction. A sentence of judicial interdiction is pronounced, either in an action at the instance of the prodigal's heir, or his next of kin: or *ex proprio motu* of the judge, during a suit in court. This kind of interdiction can only be removed by the authority of the court. Voluntary interdiction, again, is the act of the party applying; but after bond has been once executed, he cannot withdraw it by his own hand.

The person who, from being conscious of mental facility, thus lays him or herself under voluntary restraint, signs a bond, whereby the granter comes under an obligation to execute no deed which may effect heritable estate, without the consent of certain persons therein specially named. This form of interdiction may, however, be removed:—1. By a sentence of the Court of Session at Edinburgh, either on the ground that such a proceeding was originally unnecessary, or, that the party has, since the bond was executed, become *rei sui providus*, as so expressed by jurists. 2. Without judicial interference, it may be quashed by the joint act of the interdicted party and interdictors. 3. And lastly, where a quorum of interdictors is mentioned, the restraint ceases, if, by death or otherwise, the number becomes reduced below the denominated quorum. Such are the chief characteristics in reference to interdicting any facile or insane person in Scotland: notwithstanding which, I would add, that this procedure is now more rarely adopted than formerly; the course usually pursued, of late years, being the appointment by the Court at Edinburgh, of a *Curator bonis*, speaking judicially.

As Dr. Winslow is on the eve of bringing before the legal and medical profession in England, some important suggestions respecting a modification of the law relative to persons alleged to be mentally incompetent for the government of themselves and their affairs, but who cannot be pronounced to be either "insane," "lunatic," or of "unsound mind," I consider it would be out of place, if not altogether superfluous, to pursue so interesting a topic any further in these pages; more especially, as the whole question will assuredly be treated in the fullest manner by that able writer, and distinguished psychological physician. Leaving, therefore, the investigation of such forensic, yet professional matters—whatever phase they may assume—to be discussed by the above-named eminent medico-legal authority,* I now proceed to describe the different establishments visited during my recent excursion to Scotland, and first, respecting—

### 1.—The Royal Edinburgh Asylum.

This public institution for the insane is of modern construction, and although not yet completed, already contains the largest number of lunatics, compared with any other similar establishment throughout Scotland. It is situated near the village of Morningside, about a mile and a half to the south-west of Edinburgh. The position is beautiful, slightly elevated, airy, and salubrious. It has a southern exposure, with the Braid and Pentland hills in front, but at a considerable distance, whereby the prospect enjoyed from the main building, as also from the garden and adjoining precincts, is really splendid, and only surpassed by that of Gart-navel Asylum, near Glasgow, and of Illnau, in the Grand Duchy of Baden: amongst all the public institutions for lunatic patients which I have ever visited, whether in Great Britain, or on the Continent. The grounds are extensive, being about forty acres, in which horticultural and agricultural occupations are carried out extensively by the inmates.

The asylum possesses a bowling-green, cricket-ground, and curling-pond, which is now being considerably enlarged. There is an extensive piggery, designed by a former patient, which is really a model for imitation, and where, in consequence of the superior feeding, unusual cleanliness of all the pigsties, as also the great attention paid to other requisites, some of the best hogs are here reared throughout this district of Scotland. In addition to being placed in a healthy, open situation, Morningside has an ample supply of water—so essential in all establishments for the insane—and likewise a good well on the premises, for special purposes. The general form of the chief building is that of the letter H, having the kitchen and offices behind; whilst, farther off, are numerous workshops for patients; and at each side, when all the proposed constructions are finished, there will be a smaller but separate building, joined by covered passages, for the accommodation of noisy and refractory inmates of both sexes: each having their own bed-rooms, dormitories, day-rooms, and separate airing-grounds. Altogether, when completed, this Asylum will be of a superior description, and, in addition to its present capabilities, will afford room for nearly 200 more patients, than it can now accommodate.

Besides the buildings here briefly described, there is also another house, or rather mansion, situated within the Asylum enclosure,

* Indeed, Dr. Winslow has already mooted the question of interdiction, at page 126 of his valuable Lettsomian Lectures.

which is entirely distinct, but where only insane ladies and gentlemen are received. At the period of my visit to Morningside, this department contained sixty private patients, whose board and lodging varied from 60*l.* to 350*l.* per annum. In short, this portion of the institution resembled in every particular a private establishment for the reception and treatment of insane patients, excepting that its pecuniary transactions did not augment the profits of individuals, the whole being under one management. In illustration of this point, as likewise to show the magnitude of its operations in reference to money matters, it may be here mentioned that the total ordinary receipts throughout the past year, as reported by the treasurer, amounted to 16,053*l.* 4*s.* 6½*d.*, whilst the whole expenditure is stated at 15,532*l.* 9*s.*, during the same period.

When I inspected the Asylum now under review, early last autumn, the aggregate population of both departments—viz., pauper and private—amounted to 556 lunatics; of whom 273 were males and 283 females. Amongst these, 48 were epileptics, 28 being males and 20 females. The dirty patients reached to 31; of whom 16 were male and 15 female inmates. Those affected with general paralysis were 10 in number; 8 being males and only 2 females. Lastly, in reference to the physical health of the entire population, it was reported in such a satisfactory condition that not more than 16 individuals were indisposed—3 being male and 13 female patients; their ailments, however, appearing nearly all of a very unimportant description. No inmate was confined by any kind of personal restraint, excepting two females, who were then placed in temporary seclusion, in consequence of their violence whilst labouring under a paroxysm of excitement. The general aspect of this establishment, therefore, seemed highly satisfactory; and I was much gratified on witnessing the order, quietude, and apparent comfort of its numerous residents: notwithstanding the hopeless nature of many of their mental maladies.

During the past year, 212 new patients were admitted; of whom 98 were male and 114 female lunatics. The numbers discharged cured being 28 males and 66 females, or 94 in all; hence it appears that the recoveries reached a ratio of 44·3 per cent. to the total admissions. Fifty-one patients died during the year, 24 being males, and 27 females, which gives the proportion of 9·2 per cent. to the average population; this mortality being, it is important to mention, rather less than in any previous year since first opening the pauper department of this Asylum. Such result is the more satisfactory, seeing cholera prevailed in the neighbourhood during great part of last year, but without a single case of that malady having occurred throughout the establishment: notwithstanding two epidemics actually appeared within its walls—namely, diarrhœa and influenza.

Amongst the 212 new patients received into this Asylum during 1854, the following were the principal forms of disease which they exhibited at their admission. Acute mania was recognised in 50 cases; monomania of various types affected 47 individuals; 30 were classed under the head of dementia; 29 under melancholia; 16 were examples of moral insanity; whilst 14 were cases of general paralysis, all of whom, it should be specially noticed, being male patients.

Respecting the latter form of mental malady, which has attracted so much attention in France, and is now often noticed by British medical practitioners, I would beg to transcribe the subjoined interesting observations contained in Dr. Skae's valuable "Annual Report," read at a meeting of the contributors held last February,—viz.:

"The number of cases of that most hopeless and deplorable malady, general paralysis, is nearly double that of the previous year. Of the fourteen sufferers from this fatal disease, two only laboured under melancholy; to all the others, although sinking slowly but perceptibly under a gradually progressive paralysis of mind and body,—to most of them even when the speech was inarticulate, and the power of locomotion nearly gone, the external world continued bright with visions of wealth, and power, and beauty, which were all their own. Even in the midst of the most extravagant delusions of all kinds, the passing events of public interest helped to dress up the pageant. One was busy fighting the Russians—another was aide-de-camp to Sir Colin Campbell, and about to walk to the Crimea—another had already taken St. Petersburg, and captured the Emperor; a fourth offered cheques upon Lord Palmerston for sums of money of fabulous amount, and was in daily expectation of his lordship's carriage to take him up to London; another spoke garrulously what he imagined to be a variety of foreign languages; whilst another was now lieutenant-general of Scotland, and anon general of India,—now the greatest statesman and the most renowned warrior alive, and again the universal king of the earth and the Almighty himself. Two of the cases were ascribed to falls from a height, several of them to intemperate habits, and the others to over-worked minds, anxiety, and excitement."

With reference to the causes assigned as chiefly instrumental in producing mental disease in those admitted, it may be mentioned, on the same indubitable authority, that intemperance figured, as heretofore, the most frequent cause of insanity: a seventh, or actually 33 cases out of the total 212 admissions being of that category; two-thirds of whom were male, and one-third female delinquents—all slaves of drunkenness! Dr. Skae next observes, in reference to causes, that—

"Griefs, anxieties, and distress occasioned by reverses of fortune and domestic afflictions, come next in order as the most frequent causes of this malady. The case ascribed to imprisonment occurred to a young lad who stole under the urgent cravings of hunger, and became insane two days after he was committed to prison. In one of the cases ascribed to epilepsy, the symptoms of insanity were developed under the influence of mesmerism, which was being employed for the purpose of curing the epilepsy. Of the puerperal cases, one was ascribed to the use of chloroform during delivery; but of all the cases of puerperal insanity admitted into the Asylum since the introduction of chloroform into medical practice, amounting to 44, this is the first and only one to which this anæsthetic had been administered during labour."

The latter fact respecting puerperal insanity is highly important, not only as indicating the rarity of cases where chloroform was employed during child-birth, but that, in the single instance where the above often dangerous anæsthetic was administered to relieve labour pains, the patient became subsequently insane. If the whole truth were always known, similar results from the use of chloroform, in so natural a process as parturition, would be found more common, I believe, than some proselytes for its use will perhaps willingly admit, or could even imagine.

At this Asylum, one great object constantly kept in view during the treatment of insane patients, especially throughout the pauper department, is to occupy or amuse the inmates in the way which may be deemed most advisable. Consequently, many are employed daily in various trades and occupations, such as tailors, shoemakers, smiths, carpenters, and so forth. Working in the garden and adjoining fields, for males, and in the washing-house or laundry, for females, may be mentioned as the most beneficial, as also having the greatest repute amongst the different modes of employment. Upon an average, about 300 inmates are usually so occupied. The patients likewise enjoy all the customary means of recreation and healthy amusement, which are now deemed essential in every well-regulated institution for the insane. Frequent walking parties, daily drives, occasional excursions to the neighbouring hills, or pic-nic meetings, are permitted to inmates. Games of bowls, quoits, cricket, and curling on the ice in winter, are common amusements. Besides these, in-door recreations, such as billiards, bagatelle, and some other games, are never-failing attractions; whilst concerts, evening parties, and a regular weekly ball every Wednesday—where both sexes meet to enjoy the healthy, exhilarating influence of active exercise and music—must not be overlooked. I received a kind invitation to be present at one of these weekly re-unions; but unfortunately my limited time, and other engagements, did not permit accepting that gratification: which proves, not only a source of much pleasure to those insane persons who participate therein, but it likewise benefits many of them, by the discipline thus imposed upon their behaviour.

An interesting feature in the management of this public asylum must also be specially noticed—namely, the "Morningside Mirror," which periodical has now been regularly published during nearly ten years. It is chiefly composed by inmates, and printed at the Royal Asylum Press; the profits being devoted to the patients' reading-room. As a specimen of the matter supplied to readers, whether insane or otherwise, of this literary journal, the following extract from a recent number may be here transcribed. It was entitled the

"MONTHLY RETROSPECT.

"Notwithstanding the heavy rains of late, there have been some exciting amusements out of doors, the players of cricket, bowls, and quoits taking advantage of every sunny blink to make a 'sortie' in force, to have a throw at their favourite game, while small parties of skirmishers, making sudden attacks on the outer-works of peas and beans were often visible, and had sometimes to make a hasty retreat on the 'staff' approaching. Since the destruction of Strawberry-hill, Gooseberry-lane has become the chief object of assault, and although a small handful only may be carried off prisoners at a time, still, by perseverance and repeated attacks, they must ultimately be reduced, numerous though they be at present.

"We have again to acknowledge the kindness of the instrumental musicians belonging to Messrs. Nelson's printing establishment, for their recent visit; but we have again to regret also that the weather, though not so wet, was rather uninviting, and prevented numbers from perambulating in the vicinity of the band, which would have greatly enlivened the scene. It is hoped, however, that before the season is over, there may be a grand gala day, with cricket, bowling, promenading, &c., with the band in the centre, playing in the usually enlivening manner, while we hope also to hear at intervals across the lake the warlike strains of the bagpipes. These on the 'pibroch sounding, sounding,' we think, with other martial airs, must become the favourites while the spirit of war remains in the ascendant. It is pleasing to see that on the Wednesdays, the ball-room is occasionally visited by some eminent professional musicians, such as Mr. Wallace and Sons, Mr. Mackenzie and Brothers, with a number of others, whose appearance is hailed as a guarantee of something extra instrumentally. The vocal department can never be at a loss while the Misses M'Pherson continue to come out. The younger sister, whom we shall here designate 'Mattie'—she having appeared with us in that character—made her vocal *debût* last night, and 'came out strong' and beautifully in 'Ye banks and braes.' We are much indebted to Mr. E. Drummond, in the dramatical line, we might say personally so, as he has occasionally studied parts and dialogues to oblige, and has shown himself well up in the characters on very short notice. While he continues to lend his valuable aid, it is intended to get up a series of comic dialogues, with an occasional effort at something more lofty, in the tragic walk, by way of a change."

The medical staff at Morningside consists of one consulting physician, Dr. Gillespie: a resident physician, Dr. Skae: and two assistants—viz., Drs. Howden and M'Culloch; to the former of whom I feel especially indebted for the courtesy with which he accompanied me, in the absence of Dr. Skae—then on leave,—throughout the entire establishment, when he gave me much valuable information.

### Glasgow Royal Asylum.

This establishment for lunatics—situated at Gart-navel, about four miles north-west of Glasgow—has only been recently constructed, to replace the old Asylum located within that city: but which has now been converted into a poor-house. The situation it occupies is rather elevated, open, airy, and enjoys an extensive prospect on every side, embracing the Grampians, Ben Lomond, with the banks and rising grounds bounding the Clyde. Indeed, the locality of this Asylum really seemed one of the finest possessed by almost any public building in Scotland; and on first approaching its precincts, the structure externally, and the elevation altogether, remind the spectator much of Windsor Castle: especially when passing near the main entrance.

Although superior in respect of position, and equally salubrious, when compared with the Asylum previously described, nevertheless, in the general plan and some interior arrangements, it appeared inferior, according to my judgment; whilst, in regard to the supply of water, Gart-navel is occasionally defective, particularly during dry weather. However, so soon as the gigantic scheme of supplying that essential element to the city of Glasgow, by conveying it from Loch Katrine, shall be achieved, it is then expected, the quantity of water will always be sufficiently plentiful for every purpose.

Like that at Morningside, this public institution also receives both pauper and private patients within its walls; each division being, however, quite distinct, although the buildings adjoin. At the period of my visit to Gart-navel, there were 85 private lunatic inmates of both sexes, who paid from 15*s.* and 1*l.* 1*s.* to 4*l.* 4*s.* per week: with one at 5*l.* 5*s.*, who had consequently special accommodation. The total pauper patients, whose board averages from 8*s.* 6*d.* to 9*s.* per week, amounted to 296 individuals; hence giving an aggregate population of 381 lunatics, both sexes included, of whom 199 were males, and 182 females: thereby making the former most numerous; which coincidence is seldom observed in public asylums for the insane. No restraint exists at this Asylum, in which the strait-waistcoat is unknown. The patients appeared quiet and cleanly; many were working in the gardens and at various occupations; whilst I remarked, with much satisfaction, that the dormitories seemed more lofty and better adapted as sleeping-rooms, than many similar apartments met with in various other establishments of this description. Amongst the female pauper patients, 118 were employed out of the 143 then resident; 6 were sick or indisposed, and one was in seclusion. Of the 153 pauper male lunatics, 101 were occupied in some kind of manual employment; only one was confined by sickness; and not a single individual appeared in any way restrained. These facts consequently speak most satisfactorily, not only respecting the physical health of every inmate, but they likewise bear ample testimony in favour of the judicious management exercised throughout this establishment.

During the past year, 240 new patients were admitted, of whom 123 were male and 117 female lunatics, thereby giving a greater relative proportion of the former over the latter sex; but which preponderance is not accidental, the same circumstance having been very generally observed, in the past history of this institution, ever since its first opening, fourteen years ago. Of those admitted, 107 were affected with mania, 83 exhibited monomania, including melancholia, and 50 laboured under dementia: which form of mental disease is by no means rare among the poor of Glasgow, and seems evidently induced by the privations and miseries, to which many of that class are exposed. The number of patients dismissed cured, during the same period, were 60 males and 56 females, making a total of 116 cases, or about 48 per cent., compared with the admissions: whilst the deaths amounted to 62, comprising 32 male and 30 female patients, or 24 per cent. for both, the sexes being nearly equal in mortality. Amongst the assigned causes producing insanity in the patients admitted, the intemperate use of alcoholic liquors stands forward pre-eminent; 45, or about a fifth of the entire admissions, being attributed to this cause, 27 of whom were male and 18 female victims.

Upon this too frequent cause of mental derangement and other evils, throughout Scotland, the following judicious observations made by Dr. Mackintosh, the able physician-superintendent of the Glasgow Asylum, merit record and mature consideration. They are extracted from his last "Annual Report," which, like previous similar documents, deserve being read by all who take an interest in the management of institutions for the insane. The writer observes on this sad but important subject:—

"It is a mistake, however, to suppose that intemperance is, in every instance where it exists, the cause of the attack; for there are cases in which it is clearly evident that the pernicious habit is the result of, or is consequent upon, the mental derangement. In a considerable number of instances, it was ascertained that one or other of the parents of the patients (I speak of patients generally) had been addicted to intemperance. It was not, however, clearly ascertained in any instance that their habits had been such previous to the birth of their offspring; so that although it appears that this vice is indulged in by no means rarely by the parents of those who become insane, it is not proved by our investigations this year that the intemperance of the parents produced a predisposition in the children; it is nevertheless very likely, but this is a subject we cannot pursue farther here. Our inquiries, however, show that the children of parents having a proclivity to intemperance, and, at one period or other of their lives, actually of intemperate habits, are apt to become insane; that where the mental or physical organization of the parent is favourable to the development of intemperate habits, the offspring have a predisposition to insanity. That the children of drunken parents are apt to become intemperate is well known, and may be explained possibly by reference to the influence of example alone. But it is not of this I speak now, but of intemperance in the parent as a cause of insanity in the offspring."

Respecting general paralysis, which now so deservedly attracts the special notice of all psychological physicians, it appears 15 cases—general or partial—existed, of whom 10 occurred in male and 5 in female inmates. In reference to this intractable malady, the same experienced authority observes, with much truth, upon the cases under treatment:—

"One boasts of his amazing prowess, and that he can perform the most herculean feats; another fancies that he is possessed of enormous riches, and property of untold value; that he is the Supreme Being, a king, and the like. Voracity of appetite, and a restless activity, are not unfrequently found among patients of this class. The number of males here affected with paralysis is just double that of females. One case had been preceded by an attack of chorea; and in another case, a brother of the patient suffered from the same affection. In one, the attack was consequent upon, and was supposed to be caused by, the sudden healing up of old ulcers upon the legs. In another case, general blood-letting had been twice employed previous to the admission of the patient, and, according to the statement of the relatives, with a decidedly injurious effect. One fancies he is a king, and attaches 'Rex' to his signature; another is a Russian diplomatic agent; one says that she is the true Messiah, and denounces the greater part of the Old and New Testaments as a fabrication and imposture; another is a great Evangelist; a third has been dead and has come alive again, and believes that the millennium has begun, and that she has been inspired and commissioned to convert the world; some accuse themselves of having committed enormous crimes; others, by far the greater number, are the victims of plots and conspiracies; some profess to be the special favourites of Heaven, and to have divine revelations, while others are possessed of devils; one fancies that part of her body is made of glass, and dreads being touched; another that she is the

Evil One, and that her skin has become black; some are tormented in one way or other by means of gas; others are under mesmeric or electrical influences."

During the past year, 116 patients were discharged cured; many of whom, however, appeared so hopeless of benefit on admission, that it was scarcely expected they would ever have become convalescent. If the proportion of cases be calculated according to the total number admitted, the per centage hence amounts to 48·3: and as 62 died, the deaths upon the same ratio reached 22·14 per cent. Amongst the cases ending fatally, 32 were males and 30 females; but although the mortality in each sex was recently nearly equal: still, past experience of this Asylum proves the proportion of deaths ranged considerably higher in male, than female lunatics. Of the chief causes of death, it may be stated, as showing the physical diseases under which the patients succumbed, that 13 died from paralysis, 11 through exhaustion, 9 by diarrhœa, 9 were phthisis, 3 originated in cardiac affections, 3 by apoplexy or cerebral disease, and 3 followed pneumonia or peripneumonia.

Dr. Mackintosh being so well known as an experienced practitioner in mental maladies, it seems almost superfluous to allude to the treatment, whether medical, physical, or moral, pursued in this Asylum. Nevertheless, the subjoined paragraph from the recent official Report of that accomplished physician seems worthy of being quoted on the present occasion, since it conveys to readers a correct notion of the general views entertained. He says:—

"The use of warm baths has been cautiously extended, and long-continued immersion practised, in some instances with a marked beneficial sedative effect, in cases in which there was much excitement. Although general blood-letting has now been almost if not entirely discarded in the treatment of the insane, recourse is still occasionally had to the local abstraction of blood, either by means of leeches applied to the head or cupping-glasses to the nape of the neck. The great mass of the patients, however, cannot bear depletion in any form, but the reverse; and therefore, from the time of admission, stimulating nourishment, with alcoholic agents, as they are clearly indicated, are freely given, and with beneficial results. When in Forfarshire, I found, in my practice there, that the insane could bear any sort of reduction, whether by means of blood-letting or medicine, very much better than they can do here. The patients in this quarter are more exhausted, more reduced every way on admission."

Amusements and occupations of various kinds are assiduously pursued at the Gart-navel institution. Four subscriptions to the libraries of Glasgow are paid, so that the patients may possess the newest books and periodicals for their recreation, if not instruction. There is a printing-press in the Asylum, which continues to be used by inmates. They compose the articles, put them in type, and then act as pressmen in throwing off the printed copies: whilst a great many schedules, and similar sheets have been thus printed, as also part of Shakspeare, with original introductory notes by a resident lunatic. To one patient—a profound melancholic—marble, and the necessary implements were procured, whereby he was induced to commence work; and before leaving the institution convalescent, this party had executed several sculptures in a very superior manner; thus showing the utility of occupying maniacs in a way their natural genius often indicates.

The medical staff of this Royal Asylum consists of Dr. Mackintosh, physician-superintendent, with Drs. Robertson and Ferguson as assistants; besides Dr. Fleming, of Glasgow, who is surgeon to the institution, but non-resident. There being thus three resident medical officers; it hence follows that, for every 127 patients one special medical attendant has been provided, which constitutes a much higher proportion than prevails in many other similar establishments; consequently such an arrangement deserves particular notice, as also high commendation.

*(To be continued.)*

---

